# Primary Tumor Sidedness Predicts Bevacizumab Benefit in Metastatic Colorectal Cancer Patients

**DOI:** 10.3389/fonc.2019.00723

**Published:** 2019-08-14

**Authors:** Xia-Hong You, Can Wen, Zi-Jin Xia, Fan Sun, Yao Li, Wei Wang, Zhou Fang, Qing-Gen Chen, Lei Zhang, Yu-Huang Jiang, Xiao-Zhong Wang, Hou-Qun Ying, Zhen Zong

**Affiliations:** ^1^Department of Clinical Laboratory, Jiangxi Province Key Laboratory of Laboratory Medicine, The Second Affiliated Hospital of Nanchang University, Nanchang, China; ^2^Department of Clinical Laboratory, Jiangxi Cancer Hospital, Nanchang, China; ^3^Jiangxi Medical College, Nanchang University, Nanchang, China; ^4^Department of Nuclear Medicine, The Second Affiliated Hospital of Nanchang University, Nanchang, China; ^5^Department of Gastrointestinal Surgery, The Second Affiliated Hospital of Nanchang University, Nanchang, China

**Keywords:** primary tumor sidedness, bevacizumab, mCRC, prognosis, survival

## Abstract

The emerging debate between primary tumor location and clinical outcome of bevacizumab treated metastatic colorectal cancer (mCRC) continues. The aim of the present study is to investigate the association between the primary tumor location and clinical outcome of 115 mCRC patients receiving bevacizumab based treatment. A meta-analysis including 21 studies was carried out to confirm the conclusion. In our prospective study, we found that right-sided mCRC commonly occurred in older cases (*p* = 0.03) with multiple-site metastasis (*p* = 0.03). Progression-free survival (PFS) of the left-sided patients undergoing bevacizumab plus a FOLFIRI regimen was superior to the right-sided cases (*p* = 0.03, crude HR = 0.31, 95%CI = 0.11–0.87; adjusted HR = 0.21, 95%CI = 0.06–0.66). The meta-analysis confirmed that efficacy of bevacizumab-based treatment in left-sided mCRC patients was better than the right-sided cases in the overall population (*P*_h_ = 0.24, combined OR = 1.36, 95%CI = 1.07–1.72), *RAS*/*BRAF* wild-type (*P*_h_ = 0.19, combined OR = 1.66, 95%CI = 1.17–2.34), clinical trial (*P*_h_ = 0.23, combined OR = 1.42, 95%CI = 1.07–1.88), Caucasian population (*P*_h_ = 0.18, combined OR = 1.37, 95%CI = 1.02–1.85) and first-line (*P*_h_ = 0.19, combined OR = 1.48, 95%CI = 1.13–1.96) subgroups. Improved survival of bevacizumab plus chemotherapy treated left-sided mCRC patients was observed in the overall population [*P*_h_ < 0.01, combined MSR = 1.09, 95%CI = 1.00–1.18 for PFS; *P*_h_ < 0.01, combined MSR = 1.24, 95%CI = 1.13–1.36 for overall survival (OS)], especially in the *RAS/BRAF* wild-type (*P*_h_ = 0.09, combined MSR = 1.10, 95%CI = 1.03–1.19 for PFS; *P*_h_ = 0.02, combined MSR = 1.34, 95%CI = 1.21–1.49 for OS). These findings indicate that primary tumor sidedness can predict clinical outcome of bevacizumab-treated *RAS/BRAF* wild-type mCRC patients and the left-sided patients may benefit more from bevacizumab plus FOLFIRI.

## Introduction

Colorectal cancer (CRC) is the fourth most commonly diagnosed malignancy and the second leading cause of cancer-related death worldwide (1). Due to the invasiveness of digestive endoscopy and limited sensitivity of fecal immunochemical tests, the majority of new cases are usually diagnosed at the advanced stages of the disease (2). In addition to palliative surgery and radiochemotherapy, anti-epidermal growth factor receptor monoclonal antibody (anti-EGFR mAb), and anti-vascular endothelial growth factor (anti-VEGF) mAb have been used to prolong the survival of metastatic CRC (mCRC) patients (3). Nevertheless, clinical outcomes of the two inhibitor managed mCRC patients remain unsatisfactory in the clinic, with evidence showing that objective response rates (ORRs) and median progression-free survival (PFS) of cetuximab or bevacizumab-based chemotherapy are 59.6% and 10.5 months in *KRAS*-wild patients, and 62.1% and 9.5 months in the overall patient population (4, 5), respectively. Thus, robust prognostic and predictive factors which can more precisely stratify suitable patients to receive the optimal biological therapy may help to improve the clinical efficacy and outcome.

Recently, accumulating evidence has shown the significant differences in clinical characteristics, anatomic structure, embryological origin, and the genetic mutation profile between left- and right-sided CRC (6). The role of primary tumor localization has extensively increased attention, for its impact on response to biological therapy and the survival of the patient (7–11). The latest clinical trials and meta-analyses confirmed that *KRAS-*wild patients with left-sided mCRC derived great benefit from EGFR-inhibitor contained treatment (12, 13), and the inhibitor has been recommended as a first-line therapeutic treatment for patients in the 2017 National Comprehensive Cancer Network guideline (14).

Nowadays, several studies reported the involvement of primary tumor sidedness in clinical efficacy and prognosis of refractory mCRC individuals with treatment of bevacizumab-containing chemotherapy (15–17). Nevertheless, no consensus of the association between them has been achieved and its controversy still continues (18). FIRE-3, AVF2107g, and NO16966 trials show that the efficacy of bevacizumab is independent of primary tumor sidedness in mCRC patients (13, 19). On the contrary, other trials and retrospective studies imply a significantly different survival in two-sided patients undergoing bevacizumab-based therapy (15–17, 20). Studies performed by Aljehani and Boisen et al. reported that two-sided patients could benefit from bevacizumab and chemotherapy, whereas a right-sided cancer origin was associated with poor response and high mortality among patients undergoing bevacizumab, compared to left-sided cases (7, 20).

In the present study, a prospective study including 115 bevacizumab-treated mCRC patients and a meta-analysis containing 13 clinical trials and eight non-clinical trials was carried out to comprehensively understand the role of primary tumor location in the effectiveness of bevacizumab in mCRC patients.

## Materials and Methods

### Eligible Population

To investigate the involvement of primary tumor sidedness in the prognosis of bevacizumab treated mCRC patients, we prospectively screened eligible mCRC patients at the Second Hospital of Nanchang University and Jiangxi Cancer Hospital from August 2012 to August of 2015. The inclusion and exclusion criteria are as follows: (1) all of the included patients were first confirmed as mCRC through both imaging and pathological examination; (2) all enrolled patients received bevacizumab and standard chemotherapy; (3) all eligible cases were willing to participate in the study and written informed consent was obtained from all enrolled patients. Those without definite diagnosis or bevacizumab-based therapy were excluded from the study. Tumors located at the caecum to the transverse colon were defined as right-sided CRC, and those located within the splenic flexure, and beyond were considered as left-sided. The present study was approved by the Medical Ethics Committees of the Second Affiliated Hospital of Nanchang University and Jiangxi Cancer Hospital, respectively.

### Follow-Up and Clinical Response Evaluation

We performed follow-ups each 3 months in the first 2 years, and each 6 months in the third year to achieve (PFS) and overall survival (OS), with a deadline of August 2018. The time since the enrolment day to tumor enlargement or new metastases and death or its deadline were defined as PFS and OS, respectively. During the same time, clinical efficacy of bevacizumab and adjuvant chemotherapy was assessed after 3 months of regimen usage according to the response evaluation criteria in solid tumors (RECIST) guideline (version 1.1). The evaluated responses were defined as complete response (CR), partial response (PR), stable disease (SD), and progressive disease (PD), respectively. We calculated objective response rate (ORR) according to the evaluated result.

### Relevant Study Identification and Data Extraction

In order to further understand the association between primary tumor location and bevacizumab efficacy in mCRC patients, we screened and identified eligible studies to perform a meta-analysis. A comprehensive retrieval was carried out by two investigators (X-HY and Z-JX) in PUBMED, EMBASE, and the Cochrane Library as well as the CNKI database until June 2018. The following medical search terms were selected to screen relative articles: “rectal, colon, colorectal,” “cancer, tumor, neoplasms, or carcinoma,” “sided, sidedness, side, location, localization, site,” and “prognosis, survival, outcome.” Moreover, we manually searched for additional studies by screening the references of the relevant articles, and enrolled eligible studies according to the following inclusion criteria: (1) original article reported the survival of left- and right-sided mCRC with treatment of bevacizumab and chemotherapy; (2) relevant study provided clinical characteristics, clinical response, median survival time or hazard ratio (HR) and 95% confidential interval (CI). Subsequently, two investigators (X-HY and Z-JX) independently extracted clinical characteristics (first author, publication year, region, race, study design, clinical trial, treatment, included patients, median age, gender), response and survival data. Inconsistent data was discussed with a third investigator (CW) to reach a consensus by analyzing the full-text.

### Statistics

The baseline characteristics of the included patients and the response data were presented by numbers and proportions. PFS and OS emerged as the median survival in months. The relationship between primary tumor sidedness and clinical response to bevacizumab was assessed by Pearson χ^2^ test, and odds ratio (OR) and 95%CI were selected to measure the strength between them. Kaplan-Meier curve (log-rank test) and Cox regression analysis were selected to examine survival difference between left- and right-sided mCRC cases. HR and median survival ratio (MSR) were presented to show the strength between them. Heterogeneity of eligible studies in the meta-analysis was evaluated by Q test and estimated *I*^2^, *P*_h_ < 0.1 or *I*^2^ > 50% was recognized as a significant heterogeneity between them. According to the heterogeneity test, the Z test in the fixed (*P*_h_ > 0.1) or random (*P*_h_ < 0.1) model was selected to analyze the combined effect in the meta-analysis. All of the statistics were performed using the SPSS statistical 17.0 (SPSS Inc., Chicago, IL) and Stata 11.0 software (Stata Corporation, College Station, TX), *p* < 0.05 was recognized as a statistical significance between the comparison.

## Results

In the present study, 74 left-sided mCRC patients and 41 right-sided cases were enrolled according to inclusion and exclusion criteria. The baseline characteristics are described in Table 1. The left-sided patients were significantly younger than the right-sided cases (*p* = 0.03), multiple sites metastasis (*p* = 0.03) was frequently observed in right-sided patients compared to the left-sided individuals. In addition to all of the patients that received bevacizumab and chemotherapy, 56.76% of the left-sided mCRC patients and 48.78% of the right-sided patients received palliative resection, and radiotherapy-treated left-sided cases were higher than the right-sided patient (*p* = 0.01). Due to intolerance of chemotherapy cytotoxicity effect, nine patients retired from the study and the response and survival data was only obtained from the remaining 106 cases. Among them, 30, 48, and 28 mCRC cases were evaluated as PR, SD, and PD, respectively. Disease progression was observed in 88 patients and 49 patients died, with a median PFS and OS of 9 and 21 months, respectively.

**Table 1:** The baseline characteristics of 115 mCRC patients in the present study.

**Variables**	**The total cases (*N* = 115)**	**Left-sided cases (*N* = 74)**	**Right-sided cases (*N* = 41)**	***P-*value**
Age (mean)	55	53	59	0.03
Age group, no. (%)				
≤ 60 year	75 (65.22)	54 (72.97)	21 (51.22)	0.02
>60 year	40 (34.78)	20 (27.03)	20 (48.78)	
Gender (male/female)	64/51	45/29	19/22	0.14
Smoking, No. (%)	12 (10.43)	9 (12.16)	3 (7.32)	0.42
Drinking, No. (%)	7 (6.09)	4 (5.41)	3 (7.32)	0.68
Diabetes, No. (%)	6 (5.22)	4 (5.41)	2 (4.88)	0.90
Hypertension, No. (%)	18 (15.65)	12 (16.2)	6 (14.63)	0.82
Metastasis, no. (%)				
Multiple sites	44 (38.26)	23 (31.08)	21 (51.22)	0.03
Single site	71 (61.74)	51 (68.92)	20 (48.78)	
Liver	42 (36.52)	30 (40.54)	12 (29.27)	0.23
Peritoneum	12 (10.44)	7 (9.46)	5 (12.15)	0.65
Other sites	17 (14.78)	14 (19.72)	3 (7.32)	0.08
Bevacizumab +CT, No. (%)	115 (100.00)	74 (100.00)	41 (100.00)	
FOLFOX	61 (53.00)	37 (50.00)	24 (58.50)	
FOLFIRI	28 (20.00)	21 (17.60)	7 (24.40)	-
FOLFOXIRI	23 (24.30)	13 (17.10)	10 (28.40)	
Capecitabine	3 (2.60)	3 (4.10)	0 (0.00)	
Palliative resection, No. (%)	62 (53.91)	42 (56.76)	20 (48.78)	0.41
Radiotherapy, No. (%)	21 (18.26)	19 (25.68)	2 (4.88)	0.01
Clinical response, No. (%)	106 (92.17)	70 (94.59)	36 (87.80)	
CR	0	0	0	
PR	30 (28.30)	20 (28.57)	10 (27.78)	0.43
SD	48 (45.28)	29 (41.43)	19 (52.78)	
PD	28 (26.42)	21 (30.00)	7 (19.44)	
No. of progressive cases	88 (76.52)	58 (78.38)	30 (43.17)	0.53
Median PFS (months)	9.00	9.00	8.50	
No. of dead cases	49 (42.61)	34 (45.95)	15 (36.59)	0.49
Median OS (months)	21.00	22.5	21.00	

*CT, chemotherapy; CR, complete response; PR, partial response; SD, stable disease; PD, progressive disease; PFS, progression-free survival; OS, overall survival; FOLFOX, fluorouracil, leucovorin, and oxaliplatin; FOLFIRI, fluorouracil, leucovorin, and irinotecan; FOLFOXIRI, oxaliplatin, fluorouracil, and irinotecan*.

**Figure 1:**
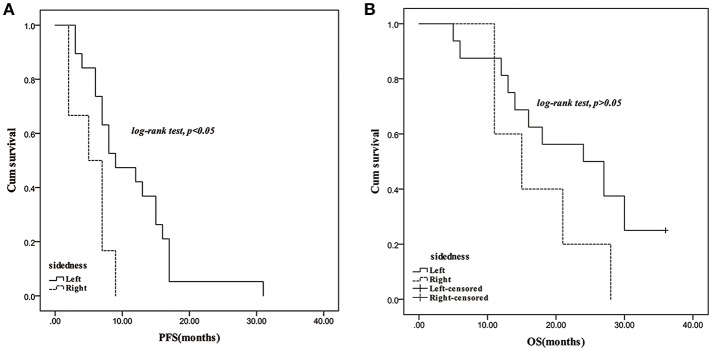
Survival comparison between right- and left-sided mCRC patients with treatment of bevacizumab plus FOLFIRI regimen. **(A)**: PFS; **(B)**: OS.

In response to bevacizumab, 28.57% of left-sided mCRC patients, and 27.78% of the right-sided cases were assessed as CR/PR, respectively. No difference of ORR was observed between the right- and left-sided cases and the two-sided patients stratified by different therapeutic regimen (Supplementary Table 1). In the follow-up period, disease progression was observed in 78.38 and 73.17% of the left- and right-sided cases, and the median PFS had no difference between them (9 vs. 8.5 months). Thirty-four left-sided patients and 15 right-sided individuals died, and the two-sided patients harbored 22.5 and 21 months of median OS, respectively. No significant PFS and OS difference was observed between the two-sided overall patients. According to the therapeutic regimen, there was no significant survival difference between the two-sided cases regardless of palliative surgery or radiotherapy. No survival difference was observed between the two-sided patients undergoing bevacizumab combined FOLFOX or FOLFOXIRI regimens. However, PFS of right-sided patients undergoing bevacizumab and a FOLFIRI regimen was significantly inferior to the left-sided cases (*p* = 0.03, crude HR = 0.31, 95%CI = 0.11–0.87; *p* = 0.01, adjusted HR = 0.21, 95%CI = 0.06–0.66) (Figure 1 and Supplementary Tables 2, 3).

**Figure 2:**
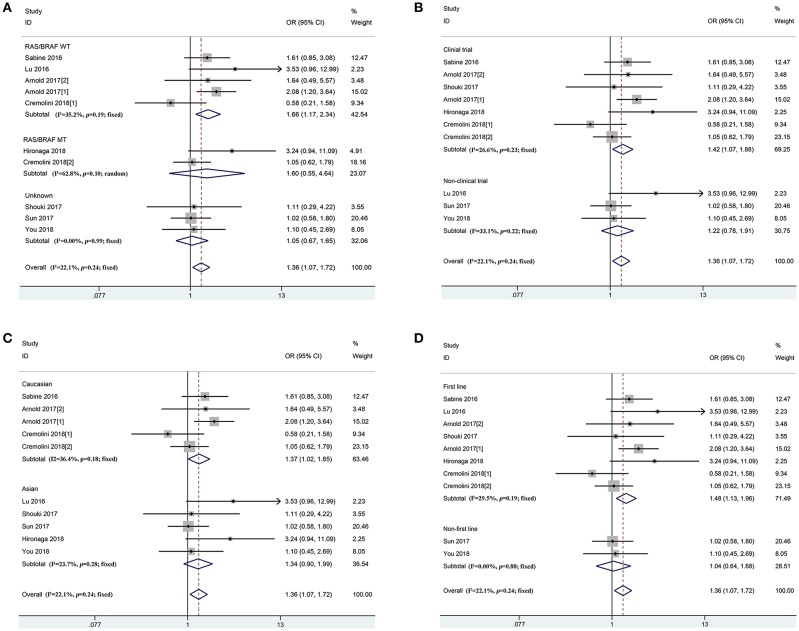
Combined effect of objective response rates (left vs. right) in the overall population and subgroup, stratified by *RAS/BRAF* status **(A)**, study design **(B)**, population **(C)**, treatment-line **(D)**.

In accordance with the inclusion criteria of eligible studies, a total of 21 studies including 4,416 patients were enrolled in the meta-analysis (12, 13, 15, 21–35) (Supplementary Figure 1). Among them, two prospective and 19 retrospective studies were included. Seventeen (11 clinical trials and 6 non-clinical trials) and four studies reported the first-line and non-first-line usage of bevacizumab and chemotherapy in mCRC cases, respectively. Moreover, 9, 16, and 19 eligible studies reported clinical efficacy of the therapeutic regimen, PFS and OS of the patients, respectively. The baseline characteristics of included studies are described in Table 2. Combined ORR of left-sided mCRC patients was superior to right-sided cases (*P*_h_ = 0.24, combined OR = 1.36, 95%CI = 1.07–1.72) (Figure 2 and Supplementary Table 4). When stratifying according to the *RAS/BRAF* status, population, study design and treatment line, we found that primary tumor sidedness was significantly associated with clinical response to bevacizumab and chemotherapy in the *RAS/BRAF* wild-type patients (*P*_h_ = 0.19, combined OR = 1.66, 95%CI = 1.17–2.34) (Figure 2A), clinical trials (*P*_h_ = 0.23, combined OR = 1.42, 95%CI = 1.07–1.88) (Figure 2B), Caucasian population (*P*_h_ = 0.18, combined OR = 1.37, 95%CI = 1.02–1.85) (Figure 2C), as well as first-line (*P*_h_ = 0.19, combined OR = 1.48, 95%CI = 1.13–1.96) (Figure 2D) subgroup, respectively.

**Table 2:** Baseline characteristics of included studies.

**Study**	**Population**	**Clinical trial**	**Treatment line**	***RAS/BRAF* status**	**Therapeutic regimen**	**Cases**	**Left-side**	**Right-side**	**Male/Female**	**Outcome**
Calvetti et al. (21)	Caucasian	Non-clinical trial	First line	Wild type	Chemotherapy + Bev	81	NA	NA	NA	OS
Tejpar et al. (13)	Caucasian	FIRE-3	First line	Wild type	Chemotherapy + Bev	199	149	50	NA	OS, PFS, ORR
Lu et al. (22)	Asian	Non-clinical trial	First line	Wild type	Chemotherapy + Bev	54	30	24	37/17	OS, PFS, ORR
He et al. (23)	Asian	Non-clinical trial	First line	Unknown	Chemotherapy + Bev	164	86	78	100/64	OS
Arnold et al. (12)	Caucasian	PEAK	First line	Wild type	Chemotherapy + Bev	68	54	14	NA	OS, PFS, ORR
Sun et al. (24)	Asian	Non-clinical trial	Non-first line	Unknown	Chemotherapy + Bev	217	138	79	120/97	OS, PFS, ORR
Houts et al. (25)	Mix	CALGB 80405	First line	Wild type	Chemotherapy + Bev	241	162	79	140/114	OS
Arnold et al. (12)	Caucasian	CALGB 80405	First line	Wild type	Chemotherapy + Bev	230	152	78	NA	PFS, ORR
Bazarbashi et al. (26)	Asian	NCT01311050	First line	Unknown	Chemotherapy + Bev	53	42	11	28/25	OS, PFS, ORR
Ulivi et al. (27)	Caucasian	NCT01878422	First line	Unknown	Chemotherapy + Bev	53	30	23	NA	OS, PFS
Arora et al. (28)	Caucasian	Phase 1 clinical trial	Non-first line	Unknown	Chemotherapy + Bev	121	86	35	85/36	OS, PFS
Demircan et al. (29)	Asian	Non-clinical trial	First line	Unknown	Chemotherapy + Bev	360	NA	NA	201/159	OS, PFS
Reinacher et al. (30)	Caucasian	AIO KRK 0207	First line	Unknown	Chemotherapy + Bev	414	NA	NA	NA	OS, PFS
Artaç et al. (31)	Asian	Non-clinical trial	First line	Wild type, Mutant type	Chemotherapy + Bev	371	270	101	228/335	OS, PFS
Loupakis et al. (32)	Mix	AVF2107g	First line	Unknown	Chemotherapy + Bev	298	195	103	NA	OS, PFS
Loupakis et al. (32)	Mix	NO16966	First line	Unknown	Chemotherapy + Bev	497	380	117	NA	OS, PFS
Cremolini et al. (15)	Caucasian	TRIBE	First line	Wild type, Mutant type	Chemotherapy + Bev	358	242	116	218/140	OS, PFS, ORR
Satake et al. (33)	Asian	JACCRO CC-11	First line	Mutant type	Chemotherapy + Bev	62	45	17	34/28	PFS, ORR
Chibaudel et al. (34)	Caucasian	DREAM	Non-first line	Wild type, Mutant type	Chemotherapy + Bev	348	250	98	NA	OS
Nakamura et al. (35)	Asian	Non-clinical trial	First line	Unknown	Chemotherapy + Bev	112	NA	NA	NA	OS
You et al.	Asian	Non-clinical trial	Non-first line	Unknown	Chemotherapy + Bev	115	74	41	64/51	OS, PFS, ORR

*Bev, bevacizumab; OS, overall survival; PFS, progression-free survival; ORR, objective response rate; NA, not available*.

**Figure 3:**
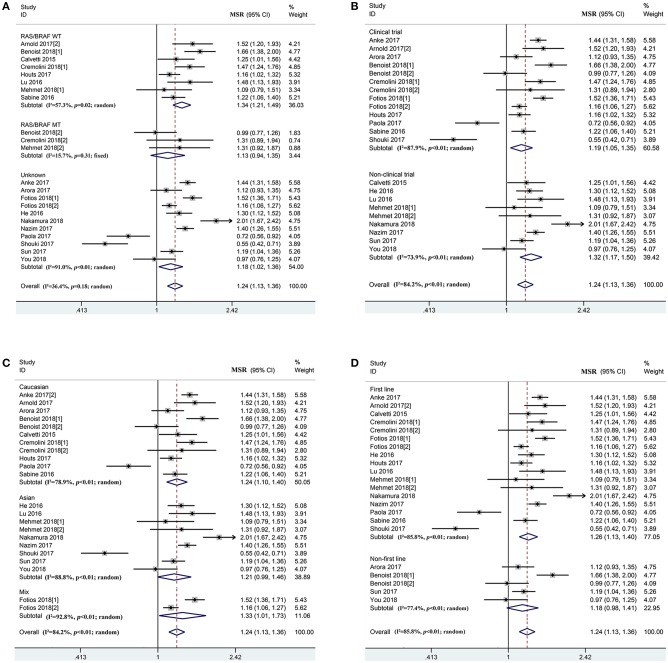
Combined effect of median overall survival ratio (left vs. right) in the overall population and subgroup, stratified by *RAS/BRAF* status **(A)**, study design **(B)**, population **(C)**, treatment-line **(D)**.

According to the prognosis of mCRC patients in the overall population, PFS (*P*_h_ < 0.01, combined MSR = 1.09, 95%CI = 1.00–1.18) and OS (*P*_h_ < 0.01, combined MSR = 1.24, 95%CI = 1.13–1.36) within left-sided mCRC patients were significantly longer than those of the right-sided cases (Figure 3 and Supplementary Figure 2, Supplementary Table 5). Moreover, compared to right-sided mCRC patients, bevacizumab-treated left-sided mCRC cases showed improved PFS in the *RAS/BRAF* wild-type (*P*_h_ = 0.09, combined MSR = 1.10, 95%CI = 1.03–1.19) (Supplementary Figure 2A), non-clinical trials (*P*_h_ = 0.12, combined MSR = 1.23, 95%CI = 1.14–1.32) (Supplementary Figure 2B), and mixed population (*P*_h_ = 0.17, combined MSR = 1.15, 95%CI = 1.08–1.23) (Supplementary Figure 2C) subgroups. In addition, primary tumor sidedness was significantly associated with improved OS in mCRC patients undergoing bevacizumab and chemotherapy regardless of the study design (Figure 3B), population (Figure 3C), and treatment line (Figure 3D), especially in *RAS/BRAF* wild-type patients (*P*_h_ = 0.02, combined MSR = 1.34, 95%CI = 1.21–1.49) (Figure 3A).

## Discussion

The impact of primary tumor location on bevacizumab plus adjuvant chemotherapy in mCRC patients remains controversial (16, 18). In this study, we found that left-sided mCRC patients could benefit more from bevacizumab plus FOLFIRI compared with its counterpart. With the large sample size, the robust results of the meta-analysis showed that clinical efficacy and survival of bevacizumab treated left-sided patients was significantly superior to right-sided patients.

1. Advanced CRC is a heterogeneous disease with a varied clinical efficacy and prognosis. Left- and right-sided diseases are reported to be distinct in clinical characteristics and mutation profiles of oncogenes and anti-oncogenes as well as clinical outcomes (36–38). Thus, common therapeutic strategies such as anti-VEGF and anti-EGFR antibody, essential pathway kinase and immune checkpoint inhibitors should be carefully selected based on the patient (39, 40). According to bevacizumab, the controversy is still undergoing with which kind of patients should to use suitably. Loupakis et al. reported that clinical outcomes of both two-sided mCRC patients were improved by treatment with bevacizumab and chemotherapy (41). In our study, we found that right-sided mCRC commonly occurred in older patients with multiple site metastasis, which is consistent with the report by Yang et al. (42). Our previous study indicated that prognosis of chemotherapy-treated right-sided mCRC patients was inferior to left-sided cases (43). Our prospective study showed that PFS of bevacizumab and FOLFIRI treated left-sided patients was significantly longer than the right-sided cases. Moreover, the meta-analysis indicated that the effect of bevacizumab-based treatment in left-sided mCRC patients was better than that of right-sided cases in the *RAS/BRAF* wild-type, clinical trial, Caucasian population, and first-line subgroups. It demonstrated that primary tumor sidedness could predict clinical efficacy and survival of mCRC patients with treatment of bevacizumab and chemotherapy, and the left-sided patients could benefit from a longer survival time from the therapeutic regimen than the right-sided cases, especially in bevacizumab, and FOLFIRI treated patients.

As we know, bevacizumab can combine with VEGF to inhibit angiogenesis. Compared to the proximal colon, VEGF was observed to be abundantly expressed in CRC in the distal colon and rectum (41, 44, 45). Moreover, chromosomal instability (CIN) was commonly observed in ~75% of left-sided patients and the right-sided cases usually harbored high microsatellite instability (MSI), a CpG island methylator phenotype (CIMP) as well as a *BRAF* mutation (46). The outcome of consensus molecular subtype (CMS) 2/4 mCRC patients, with intermediate-to-high CIN, was obviously improved following bevacizumab and chemotherapy, the CMS 1/3 patients with unstable MSI and elevated CIMP, as well as low CIN could not derive further benefits from the inhibitor (47–49). In addition, distinctive gut microbiome features were observed to vary depending on primary tumor location of CRC (6, 50, 51). The gut microbiome could modulate the response to anti-PD-1 immunotherapy and adjuvant chemotherapy in melanoma and CRC (52, 53). Microbiota has been linked to chronic inflammation. Severe inflammation was reported to associated with a poor response to bevacizumab (53) and significantly higher fibrinogen to pre-albumin ratio was detected in right-sided mCRC cases compared to its counterpart (43). The above causes may therefore help us better understand the effect and prognosis of primary tumor location in bevacizumab and chemotherapy treated mCRC patients.

To the best of our knowledge, the present study is the first to perform this meta-analysis with the largest sample size to date, to investigate the prognostic, and predictive role of primary tumor location in bevacizumab-treated mCRC patients. However, the following limitations should be addressed to understand the results of our study. First, the prospective-study design used in the present study was a small sample size, including patients from only two hospitals within the same region. This might restrict a robust conclusion in our study. Second, the *KRAS/BRAF* mutation was not detected and we did not investigate the impact of it on bevacizumab efficacy and the survival of mCRC patients.

In summary, our findings illustrate that the clinical outcome of bevacizumab treated left-sided mCRC patients is superior to right-sided patients, particularly in the wild-type *RAS*/*BRAF* subgroup and bevacizumab and FOLFIR1 treated patients. Primary tumor sidedness is an effective factor used to predict the clinical response to bevacizumab and the prognosis of the patient. Considering the limitations of our study, randomized controlled trials from multiple-regions with large sample sizes are needed to verify our results.

## Data Availability

All datasets generated for this study are included in the manuscript and/or the Supplementary Files.

## Ethics Statement

Written informed consent was obtained from each enrolled patient, and the present study was approved by Medical Ethnic Committees of the Second Affiliated Hospital of Nanchang University and Jiangxi Cancer Hospital, respectively.

## Author Contributions

X-HY selected the eligible sample in the first section, screened, and selected the eligible study in the meta-analysis and performed all the statistics. CW provided the sample resource, selected the eligible patients, and prepared the clinical characteristics of each included patient in the first section. Z-JX contributed to screen, select, and identify the eligible study, prepared the clinical and survival data, and performed the statistics in the meta-analysis. FS, YL, and WW contributed to follow-up and characteristics acquisition in the first section. ZF, Q-GC, and LZ contributed to clinical and survival data acquisition in the second section. Y-HJ contributed to check-up the data. X-ZW, H-QY, and ZZ provided the idea, established the study design, revised, and approved the manuscript.

### Conflict of Interest Statement

The authors declare that the research was conducted in the absence of any commercial or financial relationships that could be construed as a potential conflict of interest.
