# Supplementary material for: Primary Tumor Sidedness Predicts Bevacizumab Benefit in Metastatic Colorectal Cancer Patients
Source: Front Oncol. 2019 Aug 14;9:723. doi: 10.3389/fonc.2019.00723 (PMC6702298; doi:10.3389/fonc.2019.00723)
Supplement: Supplementary file 1 [file Data_Sheet_1.docx]

*Supplementary Material*

**
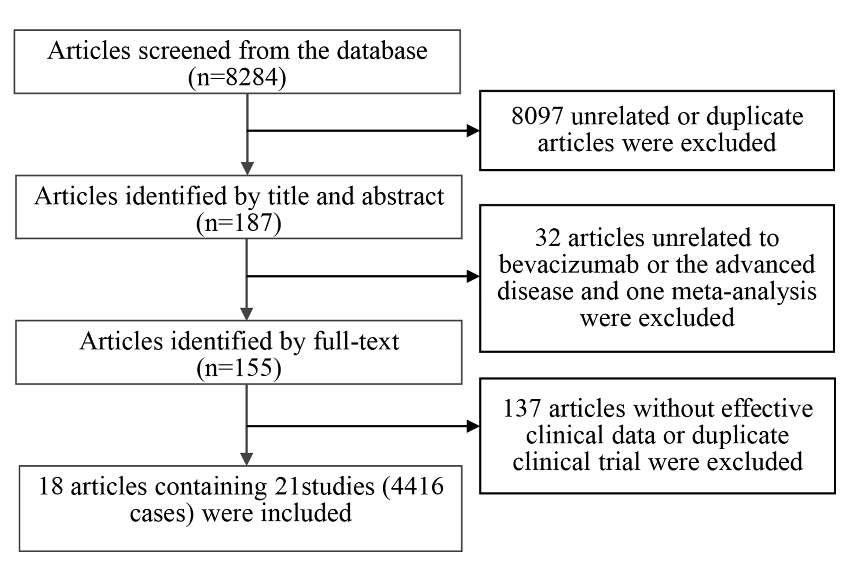
**

**Supplementary Figure 1** Flow chart of the selection procedure in the meta-analysis.

**
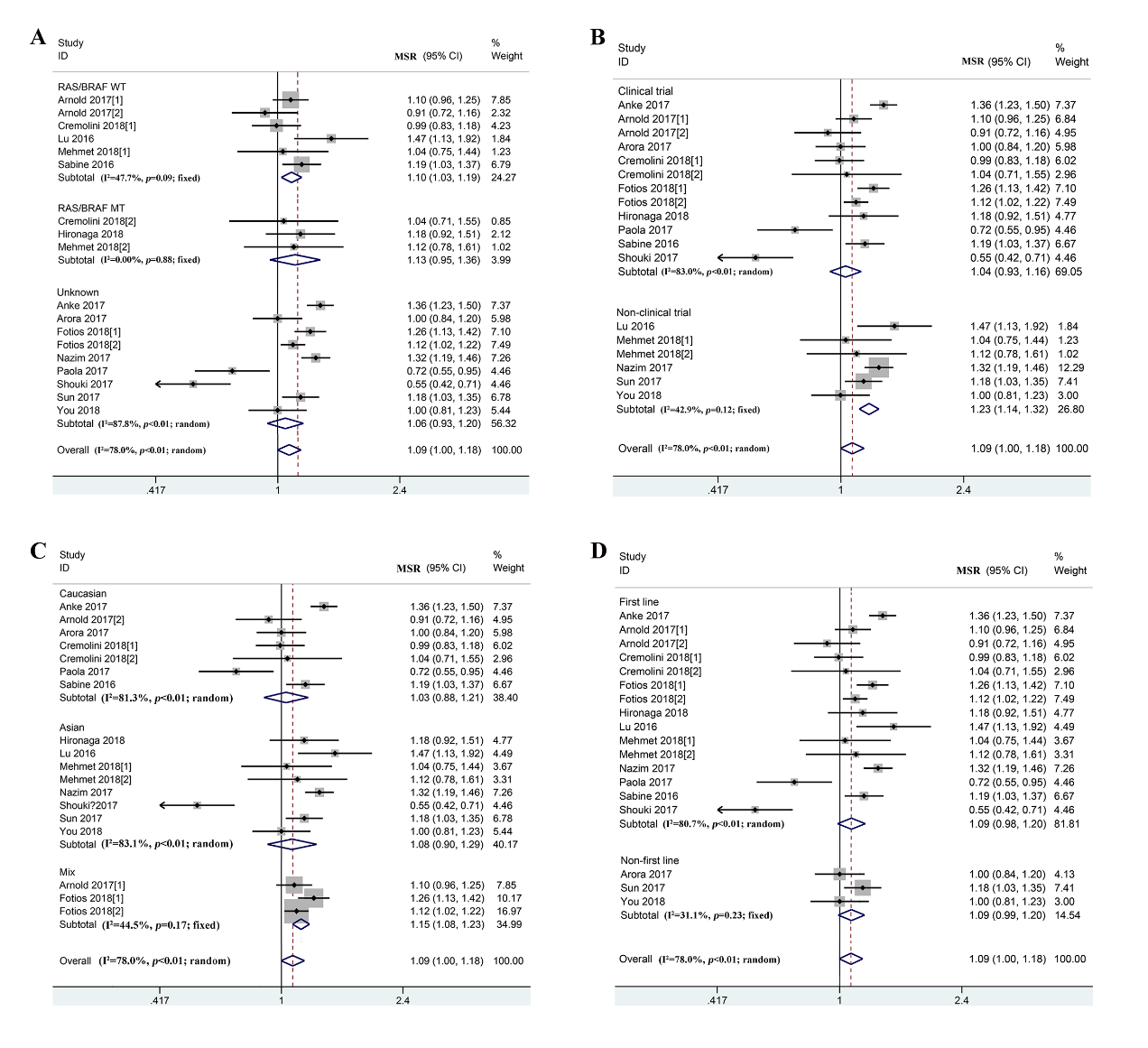
**

**Supplementary Figure 2** Combined effect of median progression-free survival ratio in the overall population and subgroup, stratified by *RAS/BRAF* status (**A**), study design (**B**), population (**C**), treatment-line (**D**).

**Supplementary Table 1** Association of primary tumor location and clinical response to bevacizumab based therapy in 106 mCRC patients.

| **Group** | **Response status** | **Primary tumor location** | | ***p*-value** | **Logistic regression** | |
| --- | --- | --- | --- | --- | --- | --- |
|  |  | **Left(n=70)** | **Right(n=36)** |  | **Crude OR(95%CI)** | **Adjusted OR (95%CI)*** |
| Overall population | CR+PR | 20(28.57%) | 10(27.78%) | 0.93 | 1.04(0.43-2.55) | 1.27(0.43-3.70) |
|  | SD+PD | 50(71.43%) | 26(72.22%) |  |  |  |
| Bevacizumab+FOLFOX | CR+PR | 10(14.29%) | 6(16.67%) | 0.96 | 0.97(0.29-3.25) | 0.72(0.16-3.35) |
|  | SD+PD | 24(34.29%) | 14(38.89%) |  |  |  |
| Bevacizumab+FOLFIRI | CR+PR | 7(10.00%) | 0(0.00%) | 0.14 | - | - |
|  | SD+PD | 13(18.57%) | 7(19.44%) |  |  |  |
| Bevacizumab+FOLFOXIRI | CR+PR | 2(2.86%) | 4(11.11%) | 0.15 | 0.23(0.03-1.68) | - |
|  | SD+PD | 11(15.71%) | 5(13.89%) |  |  |  |

**Abbreviation:** CR: complete response; PR: partial response; SD: stable disease; PD: progressive disease; OR: odds ratio; 95%CI: 95% confidential interval. FOLFOX: fluorouracil, leucovorin and oxaliplatin; FOLFIRI: fluorouracil, leucovorin and irinotecan; FOLFOXIRI: oxaliplatin, fluorouracil and irinotecan.*OR was adjusted by age, gender, smoking, drinking, diabetes, hypertension.

**Supplementary Table 2** Cox regression of primary tumor location and prognosis within 106 mCRC patients.

| **Variants** |  | | **Three years’ PFS** | |  | | **Three years’ OS** | |
| --- | --- | --- | --- | --- | --- | --- | --- | --- |
|  | **mPFS** | ***p*-value** | **HR(95%CI)** | | **mOS** | ***p*-value** | **HR(95%CI)** | |
|  |  |  | **Univariate** | **Multivariate*** |  |  | **Univariate** | **Multivariate*** |
| Age(year) | |  |  |  |  |  |  |  |
| >60 | 8.00 | 0.78 | 0.77(0.49-1.21) | 0.75(047-1.19) | 17.00 | 0.56 | 0.57(0.29-1.13) | 0.50(0.24-1.02) |
| Gender |  |  |  |  |  |  |  |  |
| Male | 8.00 | 0.31 | 1.12(0.73-1.72) | 1.08(0.67-1.74) | 19.50 | 0.44 | 0.91(0.49-1.71) | 0.83(0.39-1.75) |
| Female | 9.50 |  | 1 | 1 | 17.00 |  | 1 | 1 |
| Smoking |  |  |  |  |  |  |  |  |
| Yes | 8.00 |  | 1.25(0.62-2.50) | 1.29(0.64-2.59) | 13.50 | 0.34 | 1.43(0.63-3.24) | 1.53(0.66-3.56) |
| No | 9.00 | 0.75 | 1 | 1 | 20.00 |  | 1 | 1 |
| Drinking |  |  |  |  |  |  |  |  |
| Yes | 8.00 | 0.53 | 0.99(0.40-2.46) | 0.92(0.32-2.74) | 24.50 | 0.55 | 0.91(0.32-2.55) | 1.31(0.40-4.31) |
| No | 9.00 |  | 1 | 1 | 18.00 |  | 1 | 1 |
| Diabetes |  |  |  |  |  |  |  |  |
| Yes | 6.50 | 0.13 | 1.78(0.71-4.46) | 1.97(0.77-5.02) | 17.00 | 0.41 | 2.27(0.79-6.49) | 1.96(0.53-7.18) |
| No | 9.00 |  | 1 | 1 | 19.00 |  | 1 | 1 |
| Hypertension | |  |  |  |  |  |  |  |
| Yes | 9.00 | 0.69 | 1.39(0.78-2.47) | 1.16(0.62-2.20) | 18.00 | 0.36 | 1.82(0.88-3.74) | 2.18(1.01-4.71) |
| No | 8.00 |  | 1 | 1 | 19.00 |  | 1 | 1 |
| Palliative surgery | |  |  |  |  |  |  |  |
| Yes | 11.00 | 0.14 | 0.89(0.57-1.38) | 0.88(0.56-1.41) | 27.50 | 0.01 | 0.46(0.24-0.87) | 0.34(0.17-0.68) |
| No | 8.00 |  | 1 | 1 | 13.00 |  | 1 | 1 |
| Radiotherapy | |  |  |  |  |  |  |  |
| Yes | 11.00 | 0.14 | 0.88(0.53-1.45) | 0.78(0.46-1.32) | 28.00 | 0.44 | 1.00(0.48-2.1) | 1.12(0.46-2.75) |
| No | 8.00 |  | 1 | 1 | 17.00 |  | 1 | 1 |
| Sidedness | |  |  |  |  |  |  |  |
| Left | 9.00 | 0.96 | 0.99(0.63-1.56) | 0.94(0.59-1.50) | 18.50 | 0.95 | 1.08(0.59-1.98) | 1.19(0.63-2.22) |
| Right | 9.00 |  | 1 | 1 | 21.00 |  | 1 | 1 |

**Abbreviation:** PFS: progression-free survival; OS: overall survival; mPFS, median progression-free survival; mOS, median overall survival; HR, hazard ratio; CI, confidence interval; *p*: *p*-value of log-rank test; *HR was adjusted by age, gender, smoking, drinking, diabetes, hypertension.

**Supplementary Table 3** Univariate and multivariate analyzes of Cox regression model within different chemotherapy regimens (Left vs Right).

| **Group** | **3-y progression-free Survival** | | | |  | **3-y Overall Survival** | | | |
| --- | --- | --- | --- | --- | --- | --- | --- | --- | --- |
|  | **Univariate** | | **Multivariate** | |  | **Univariate** | | **Multivariate** | |
|  | **HR (95%CI)** | ***p-*value** | **HR(95%CI)*** | ***p-*value** |  | **HR (95%CI)** | ***p-*value** | **HR(95%CI)*** | ***p-*value** |
| Overall popualtion | 0.99(0.63-1.56) | 0.97 | 0.94(0.59-1.50) | 0.81 |  | 1.08(0.59-1.98) | 0.81 | 1.29(0.70-2.40) | 0.42 |
| Bevacizumab+FOLFOX | 1.23(0.64-2.38) | 0.53 | 1.23(0.63-2.39) | 0.55 |  | 2.22(0.80-6.14) | 0.12 | 2.59(0.91-7.36) | 0.06 |
| Bevacizumab+FOLFIRI | 0.31(0.11-0.87) | 0.03 | 0.21(0.06-0.66) | 0.01 |  | 0.44(0.15-1.32) | 0.14 | 0.43(0.09-1.98) | 0.28 |
| Bevacizumab+FOLFOXIRI | 1.33(0.52-3.38) | 0.55 | 1.53(0.40-5.84) | 0.54 |  | 0.52(0.16-1.72) | 0.28 | 0.41(0.10-1.61) | 0.20 |

**Abbreviation:** FOLFOX: fluorouracil, leucovorin and oxaliplatin; FOLFIRI: fluorouracil, leucovorin and irinotecan; FOLFOXIRI: oxaliplatin, fluorouracil and irinotecan; HR, hazard ratio; CI: confidence interval; *p*: *p*-value of log-rank test; *HR was adjusted by age, gender, smoking, drinking, diabetes, hypertension.

**Supplementary Table 4** Primary tumor location and therapeutic effect of chemotherapy plus bevacizumab in the present meta-analysis

| **Subgroup** | |  | **Left-sided** | | **Right-sided** | |  | **Heterogeneity test** | |  | **OR(95%CI)** | |
| --- | --- | --- | --- | --- | --- | --- | --- | --- | --- | --- | --- | --- |
|  |  | All | CR/PR | SD/PD | CR/PR | SD/PD |  | ***p*** | ***I*^2^(%)** |  | **Fixed model** | **Random model** |
| Overall population | | 1344 | 503 | 416 | 192 | 233 |  | 0.24 | 22.1 |  | **1.36(1.07-1.72)** | 1.37(1.03-1.83) |
| *RAS/BRAF* status | Wild type | 672 | 287 | 195 | 85 | 105 |  | 0.19 | 35.2 |  | **1.66(1.17-2.34)** | 1.62(1.01-2.61) |
|  | Mutant type | 296 | 119 | 69 | 61 | 47 |  | 0.10 | 62.8 |  | 1.25(0.77-2.02) | 1.60(0.55-4.64) |
|  | Unknown | 376 | 97 | 152 | 46 | 81 |  | 0.99 | 0.00 |  | 1.05(0.67-1.65) | 1.05(0.67-1.65) |
| Study design | Clinical trial | 968 | 418 | 265 | 148 | 137 |  | 0.23 | 26.6 |  | **1.42(1.07-1.88)** | 1.43(1.00-2.04) |
|  | No-clinical trial | 376 | 85 | 151 | 44 | 96 |  | 0.22 | 33.1 |  | 1.22(0.78-1.91) | 1.30(0.71-2.37) |
| Population | Caucasian | 853 | 357 | 239 | 132 | 125 |  | 0.18 | 36.4 |  | **1.37(1.02-1.85)** | 1.35(0.90-2.02) |
|  | Asian | 491 | 146 | 177 | 60 | 108 |  | 0.26 | 23.7 |  | 1.34(0.90-1.99) | 1.43(0.87-2.35) |
| Treat-line | First line | 1021 | 430 | 282 | 152 | 157 |  | 0.19 | 29.5 |  | **1.48(1.13-1.96)** | 1.51(1.06-2.17) |
|  | Non-first line | 323 | 73 | 134 | 40 | 76 |  | 0.88 | 0.00 |  | 1.04(0.64-1.68) | 1.04(0.64-1.68) |

**Abbreviation**: CR: complete response; PR: partial response; SD: stable disease; PD: progressive disease; OR: odds ratio; Bev: bevacizumab; CI, confidence interval.

**Supplementary Table 5** Primary tumor location and median survival rate of bevacizumab-treated mCRC patients in the present meta-analysis.

| **Subgroup** | | **Progression-free survival** | | | | |  | **Overall survival** | | | | |
| --- | --- | --- | --- | --- | --- | --- | --- | --- | --- | --- | --- | --- |
|  |  | **Heterogeneity test** | | | **HR and 95%CI** | |  | **Heterogeneity test** | | | **HR and 95%CI** | |
|  |  | ***p-value*** | | ***I^2^*** | **Fixed model** | **Random model** |  | ***p-value*** | | ***I^2^*** | **Fixed model** | **Random model** |
| **Overall population** | | 0.00 | 78.0 | | 1.16(1.11-1.36) | 1.09(1.00-1.18) |  | 0.00 | 88.8 | | 1.29(1.22-1.37) | **1.24(1.13-1.36)** |
| RAS/BRAF status | Wild type | 0.09 | 47.7 | | **1.10(1.03-1.19)** | 1.10(0.99-1.23) |  | 0.02 | 57.3 | | 1.32(1.23-1.40) | **1.34(1.21-1.49)** |
|  | Mutation | 0.88 | 0.0 | | 1.13(0.95-1.36) | 1.13(0.95-1.36) |  | 0.31 | 15.7 | | 1.13(0.94-1.35) | 1.14(0.93-1.39) |
|  | Unknown | 0.00 | 87.8 | | 1.17(1.12-1.23) | 1.06(0.93-1.20) |  | 0.00 | 91.0 | | 1.29(1.23-1.34) | **1.18(1.02-1.36)** |
| Study design | Clinical trial | 0.00 | 83 | | 1.13(1.08-1.18) | 1.04(0.93-1.16) |  | 0.00 | 87.9 | | 1.26(1.21-1.31) | **1.19(1.05-1.35)** |
|  | No-clinical trial | 0.12 | 42.9 | | **1.23(1.14-1.32)** | 1.20(1.08-1.34) |  | 0.00 | 73.9 | | 1.34(1.27-1.42) | **1.32(1.17-1.50)** |
| Population | Caucasian | 0.00 | 81.3 | | 1.15(1.08-1.22) | 1.03(0.88-1.21) |  | 0.00 | 78.9 | | 1.29(1.22-1.35) | **1.24(1.10-1.40)** |
|  | Asian | 0.00 | 83.1 | | 1.17(1.09-1.24) | 1.08(0.90-1.29) |  | 0.00 | 88.8 | | 1.29(1.22-1.37) | 1.21(0.99-1.46) |
|  | Mix | 0.17 | 44.5 | | **1.15(1.08-1.23)** | 1.16(1.06-1.26) |  | 0.00 | 92.8 | | 1.28(1.20-1.38) | **1.33(1.01-1.73)** |
| Treat-line | First line | 0.00 | 80.7 | | 1.17(1.12-1.21) | 1.09(0.98-1.20) |  | 0.00 | 85.8 | | 1.30(1.26-1.35) | **1.26(1.13-1.40)** |
|  | Non-first line | 0.23 | 31.1 | | 1.09(0.99-1.20) | 1.08(0.96-1.21) |  | 0.00 | 77.4 | | 1.21(1.11-1.31) | 1.18(0.98-1.41) |

**Abbreviation**: HR and 95%CI: hazard ratio (HR) and 95% confidential interval (CI)
